# Efficacy and Safety of Vericiguat in Heart Failure: A Systematic Review and Meta-Analysis

**DOI:** 10.7759/cureus.108653

**Published:** 2026-05-11

**Authors:** Abdullah Kilic, Ayushi Saxena, Bilal Khan, Rupanshu Rupanshu, Russaal S Mann, Isha Chopra

**Affiliations:** 1 Internal Medicine, Hackensack University Medical Center, Montclair, USA; 2 Medicine, California Institute of Behavioral Neurosciences Psychology, Fairfield, USA; 3 Health Sciences, Queen's University, Kingston, CAN; 4 Internal Medicine, Faculty of Medicine, St. Martinus University, Willemstad, CUW; 5 Internal Medicine, Vardhman Mahavir Medical College and Safdarjung Hospital, Delhi, IND; 6 Anesthesia, Government Medical College, Jaipur, IND

**Keywords:** cardiology, cardiology research, clinical cardiology, heart failure, heart failure with preserved ejection fraction (hfpef), hfpef, hfref, soluble guanylate cyclase stimulators, vericiguat

## Abstract

Patients with heart failure (HF) experience high rates of hospitalization and death, and outcomes are particularly unfavorable following recent clinical worsening or decompensation. Vericiguat is an oral soluble guanylate cyclase (sGC) stimulator that enhances nitric oxide-cyclic guanosine monophosphate signaling, with potential benefits for vascular tone and myocardial function. With the completion of recent randomized trials, an updated synthesis of the evidence is warranted. Therefore, we conducted a systematic review and meta-analysis to evaluate the efficacy and safety of vericiguat across heart failure phenotypes.

We searched PubMed, Embase, Web of Science, and the Cochrane Library for randomized controlled trials comparing vericiguat with placebo in chronic HF through September 2025. Five eligible trials (12,877 participants) were included. The primary efficacy outcome was a composite of cardiovascular death and first HF hospitalization. Data were pooled using a random-effects risk ratio (RR) model, and prespecified subgroup analysis was performed by HF phenotype: HF with reduced ejection fraction (HFrEF) and HF with preserved ejection fraction (HFpEF). The certainty of evidence was assessed using the Grading of Recommendations Assessment, Development and Evaluation (GRADE) framework.

Among patients with HFrEF (three trials; *n* = 11,611), vericiguat was associated with a lower risk of the primary composite outcome (RR 0.92; 95% confidence interval (CI), 0.87-0.98; *P* = 0.009). In a secondary, hypothesis-generating analysis, all-cause mortality was also lower with vericiguat (RR 0.91; 95% CI, 0.84-0.99; *P* = 0.03); this finding should be interpreted with caution because all-cause mortality was not a prespecified primary or co-primary endpoint in either pivotal Phase 3 trial. No clinical benefit was observed in patients with HFpEF. Vericiguat increased the relative risk of symptomatic hypotension (RR 1.20; 95% CI, 1.07-1.35; *P* = 0.002) but did not increase the risk of serious adverse events (RR 0.94; 95% CI, 0.89-1.00; *P* = 0.04).

Overall, moderate-certainty GRADE evidence suggests that vericiguat's benefit is concentrated in higher-risk HFrEF populations, particularly those with recent worsening. The evidence does not support its use for the primary composite outcome in the broader, stable ambulatory HFrEF population or in patients with HFpEF.

## Introduction and background

Heart failure (HF) is a heterogeneous clinical syndrome characterized by inadequate cardiac output and/or elevated filling pressures, which manifests as dyspnea, fatigue, and congestion from fluid retention [1,2]. It is a major public health problem, affecting more than 64 million people globally and accounting for substantial healthcare use and death [1,2]. Even with modern pharmacologic therapies, HF remains a common cause of hospitalization, particularly among older adults, because of episodes of worsening congestion and decompensation. Worsening HF was defined as a recent HF hospitalization or requirement for intravenous diuretics within three months. These frequent episodes are associated with impaired quality of life and premature mortality [3].

HF is classified by left ventricular ejection fraction (LVEF) into HF with reduced ejection fraction (HFrEF; LVEF ≤ 40%) and HF with preserved ejection fraction (HFpEF; LVEF ≥ 50%). A third category, HF with mildly reduced ejection fraction (HFmrEF; LVEF 41%-49%), has also been recognized [4]. Although all phenotypes carry meaningful risk, HFrEF has historically been associated with higher rates of cardiovascular death and recurrent HF hospitalization [5].

Current HF treatment guidelines recommend four main pharmacologic classes for the management of HFrEF, including angiotensin receptor-neprilysin inhibitors (ARNIs), evidence-based beta-blockers, mineralocorticoid receptor antagonists (MRAs), and sodium-glucose cotransporter-2 (SGLT2) inhibitors [2,3]. Large trials confirmed that these therapies have demonstrated significant reductions in mortality and hospitalizations [6,7]. However, even on maximal guideline-directed therapy, numerous patients - especially those with recent decompensation or advanced disease - continue to face high risks, underscoring the need for treatments that target alternative pathophysiologic pathways [8].

The nitric oxide (NO)-soluble guanylate cyclase (sGC)-cyclic guanosine monophosphate (cGMP) signaling pathway has emerged as a novel therapeutic target in cardiovascular disease due to its role in regulating vascular tone, myocardial relaxation, and cellular homeostasis [9]. HF disrupts this pathway through oxidative stress and endothelial dysfunction, leading to reduced NO bioavailability and diminished cGMP production [9]. The development of sGC stimulators, such as vericiguat, riociguat, and praliciguat, enables the direct activation of sGC to generate cGMP independent of NO, thereby enhancing myocardial and vascular function [10,11]. Unlike nitrates or phosphodiesterase-5 inhibitors, which indirectly increase cGMP by enhancing NO delivery or slowing cGMP degradation, vericiguat directly stimulates sGC independent of NO availability. This distinction is mechanistically important in the failing myocardium, where oxidative stress reduces NO bioavailability and oxidizes the heme moiety of sGC, rendering it unresponsive to endogenous NO; vericiguat binds to and stabilizes the reduced (Fe²⁺) heme-containing form of sGC, restoring downstream cGMP production even under conditions in which nitrate-based or phosphodiesterase-5 inhibitor therapies would be ineffective. Riociguat, the only other clinically available sGC stimulator, has a similar mechanism but is approved exclusively for pulmonary hypertension and has not shown a favorable risk-benefit profile in left-sided HF.

The clinical development of vericiguat has produced a complex body of evidence. The Armstrong et al., 2020a [12], a landmark Phase 3 study, demonstrated that vericiguat reduces the primary composite endpoint (cardiovascular death or HF hospitalization) in high-risk HFrEF patients with a recent worsening event, leading to regulatory approval for this specific population. The benefit observed in decompensated HFrEF created an evidence gap, as it remained unclear whether vericiguat would be effective in the broader ambulatory HFrEF population with stable chronic HF. The recently completed Butler et al. [13] trial was specifically designed to address this question. In contrast, trials involving patients with HFpEF, such as the phase 2 trial by Pieske et al. [14] and the phase 2b trial by Armstrong et al. [15], failed to demonstrate clinical benefit.

The availability of these new results necessitated a comprehensive, updated analysis. Previous meta-analyses that excluded the trial by Butler et al. [13] are now outdated and have produced conflicting results, complicating clinical decision-making. Prior meta-analyses produced conflicting results primarily because they pooled trials across different eras of background therapy, mixed phase 2 and phase 3 data, and were conducted before the trial by Butler et al. was available, thereby combining high-risk and stable populations with meaningfully different event rates. Our systematic review and meta-analysis synthesizes evidence from all five pivotal randomized, placebo-controlled vericiguat trials, providing the most complete quantitative assessment to date. Its aim was to evaluate vericiguat’s effectiveness and safety through a risk-based assessment by asking: Does vericiguat (intervention) provide better outcomes than placebo (comparator) in adult patients with chronic HF (population) in terms of major clinical outcomes (outcomes: cardiovascular death or HF hospitalization, all-cause mortality) and adverse events, according to randomized controlled trial (RCT) evidence (study design)? This analysis includes the trial by Butler et al. [13] to clarify vericiguat’s role in contemporary HF management.

## Review

Methodology

Protocol and Registration 

The review protocol was preregistered in PROSPERO (CRD420251144795). The manuscript follows the Preferred Reporting Items for Systematic Reviews and Meta-Analyses (PRISMA) 2020 statement [16]. To identify eligible studies, records were retrieved from PubMed, Embase, Web of Science, and the Cochrane Library up to September 10, 2025 (Appendix A).

Search Strategy 

The search strategies were tailored to each database, incorporating both standardized terms and open-ended phrases related to heart failure and vericiguat. Terms included: “heart failure,” “cardiac failure,” “ventricular dysfunction,” “vericiguat,” “Verquvo,” “MK-1242,” and “soluble guanylate cyclase stimulators.”

In CENTRAL, Embase, and Web of Science, the search was conducted using the terms ("heart failure" OR "cardiac failure") AND (vericiguat OR soluble guanylate cyclase stimulator), with filters applied to identify RCTs, when available.

Only studies conducted in human subjects were included. The search imposed no limits on language at the outset. Records identified for each database are listed in Table 1.

**Table 1:** The databases used and the number of papers identified from each database.

Search strategy	Database used	Identified records
“heart failure” AND vericiguat	PubMed	39
(“heart failure” OR “cardiac failure” OR “ventricular dysfunction”) AND vericiguat	Embase	61
(“heart failure” OR “cardiac failure”) AND vericiguat	Web of Science	48
(“heart failure” OR “cardiac failure”) AND vericiguat	Cochrane Library	164
Total number of records identified		312

Eligibility Criteria 

Trials qualified for inclusion if they fulfilled these conditions: (1) primary reports of a randomized controlled trial (RCT); (2) enrolled adult patients with chronic heart failure (HF); (3) compared vericiguat to placebo; (4) had a minimum treatment duration of 12 weeks; and (5) reported at least one prespecified clinical efficacy outcome (e.g., cardiovascular death or first HF hospitalization, all-cause mortality) or safety outcome (e.g., symptomatic hypotension or serious adverse events).

Trials were disqualified if they were non-randomized studies, case reports, editorials, trial-design manuscripts, and post hoc or secondary analyses. Studies not published in English and studies assessing sGC modulators other than vericiguat were also removed. 

Data Extraction and Risk of Bias Assessment

Independent screening of titles and abstracts was performed by two reviewers (AK and IC), followed by full-text review to determine final eligibility. When disagreements arose, resolution was achieved through discussion and, when necessary, adjudication by a third senior reviewer. Data from included trials were captured using a standardized, prespecified extraction template. The extracted data included trial design, baseline patient characteristics, dosing, treatment regimens, study duration and follow up period, funding source, and prespecified outcome assessment methods.

The reviewers collected data on eight specific outcomes: the cardiovascular death or first HF hospitalization (composite), all-cause mortality, cardiovascular death, first HF hospitalization, symptomatic hypotension, syncope, any adverse events, and serious adverse events. Risk of bias was assessed independently by two reviewers using the Cochrane Risk of Bias 2 (RoB 2) tool [17].

Outcomes and Statistical Analysis

The primary efficacy outcome of this meta-analysis was the composite of cardiovascular death or first HF hospitalization. Secondary efficacy outcomes included all-cause mortality, cardiovascular death, and first HF hospitalization. Key safety endpoints were symptomatic hypotension and serious adverse events. All five included trials reported binary event counts for prespecified clinical outcomes in their published results tables, enabling direct calculation of risk ratios (RRs) from numerator and denominator data without transformation. Where event counts for a specific outcome were not directly reported, they were derived from reported percentages and sample sizes. Time-to-event hazard ratios were not pooled; only binary event data were used for all meta-analytic calculations. Data were pooled using a random-effects model with inverse-variance weighting (DerSimonian-Laird method) to account for anticipated clinical heterogeneity across trials with differing patient risk profiles and follow-up durations. Prespecified subgroup analyses were conducted to evaluate the treatment effect by HF phenotype (HFrEF vs. HFpEF), and statistical analyses were performed in Review Manager (RevMan) version 5.4 (Cochrane Collaboration, London, United Kingdom), with significance evaluated using a two-sided *P*-value of 0.05.

RRs were chosen as the effect measure for all analyses for the following reasons: (1) all five included trials reported binary event counts in published results tables, enabling direct and transparent RR calculation without model-dependent assumptions; (2) for trials with comparable and relatively short follow-up durations (range: 12 weeks to 18.5 months), the RR approximates the HR when event rates are moderate; and (3) RRs are more intuitively interpretable for clinical decision-making. We acknowledge that HR-based pooling using log(HR) and standard errors would be the preferred approach for time-to-event data, and we recognize this as a methodological limitation. However, the primary trials [12,13] do not differ substantially in follow-up duration (10.8 vs. 18.5 months), and the resulting RR and HR estimates are numerically similar for the reported event rates.

For multi-arm phase 2 dose-ranging trials, the clinically relevant target-dose vericiguat arm and the placebo arm with extractable event counts were used for pooled analyses; therefore, outcome-level denominators differ from the total number of randomized participants across all included trials.

Although publication bias assessment with funnel plots was planned, fewer than 10 trials were available; therefore, funnel-plot analysis was not performed due to low statistical power and the potential for misleading results. The certainty of evidence for each primary and safety outcome was assessed using the GRADE framework and is presented in Appendix B [18-20].

It is acknowledged that the phase 2 trials [14,15,19] were not powered for hard clinical endpoints; their inclusion in sensitivity analyses and secondary pooled estimates is therefore considered exploratory, and primary efficacy conclusions are derived from the two large phase 3 trials [12,13]. Specifically for SOCRATES-REDUCED [19], cardiovascular death or HF hospitalization was not a prespecified primary endpoint; the trial was designed to evaluate dose-response effects on NT-proBNP. Event counts for the composite outcome were nonetheless reported in the published trial results and were therefore eligible for inclusion. To ensure that pooling these data did not bias the primary efficacy estimate, a sensitivity analysis restricted to the two adequately powered phase 3 outcome trials [12,13] was performed and is reported in the next section

Results

Study Selection and Characteristics

From the databases and registries searched, 312 records were identified. After removing 76 duplicates, 236 titles and abstracts were screened for relevance. A total of 211 records were excluded after initial screening. Twenty-five reports were selected for full-text review; however, 14 reports could not be retrieved because they were either not accessible or not published. To ensure transparency and address potential retrieval bias, we characterized the 14 reports that could not be retrieved for full-text screening. Based on titles and abstracts, these comprised: conference abstracts or poster presentations not indexed as standalone publications (*n* = 9); trial registry entries (ClinicalTrials.gov/WHO ICTRP) without an associated peer-reviewed publication, indicating no completed RCT had been published from these registrations (*n* = 3); and two records identified as duplicates of already-retrieved reports upon closer inspection. None of the 14 non-retrieved records appeared to represent a completed, peer-reviewed RCT of vericiguat versus placebo in patients with chronic HF. Eleven reports were reviewed in full text, and six studies were excluded after full-text assessment because they evaluated an sGC agent other than vericiguat (e.g., riociguat or praliciguat; n = 2) or patient populations that did not meet the inclusion criteria (e.g., those with pulmonary hypertension without chronic HF; n = 2). One report was excluded because it did not provide the prespecified clinical endpoints, and one was excluded because the follow-up period was shorter than 12 weeks.

**Figure 1:**
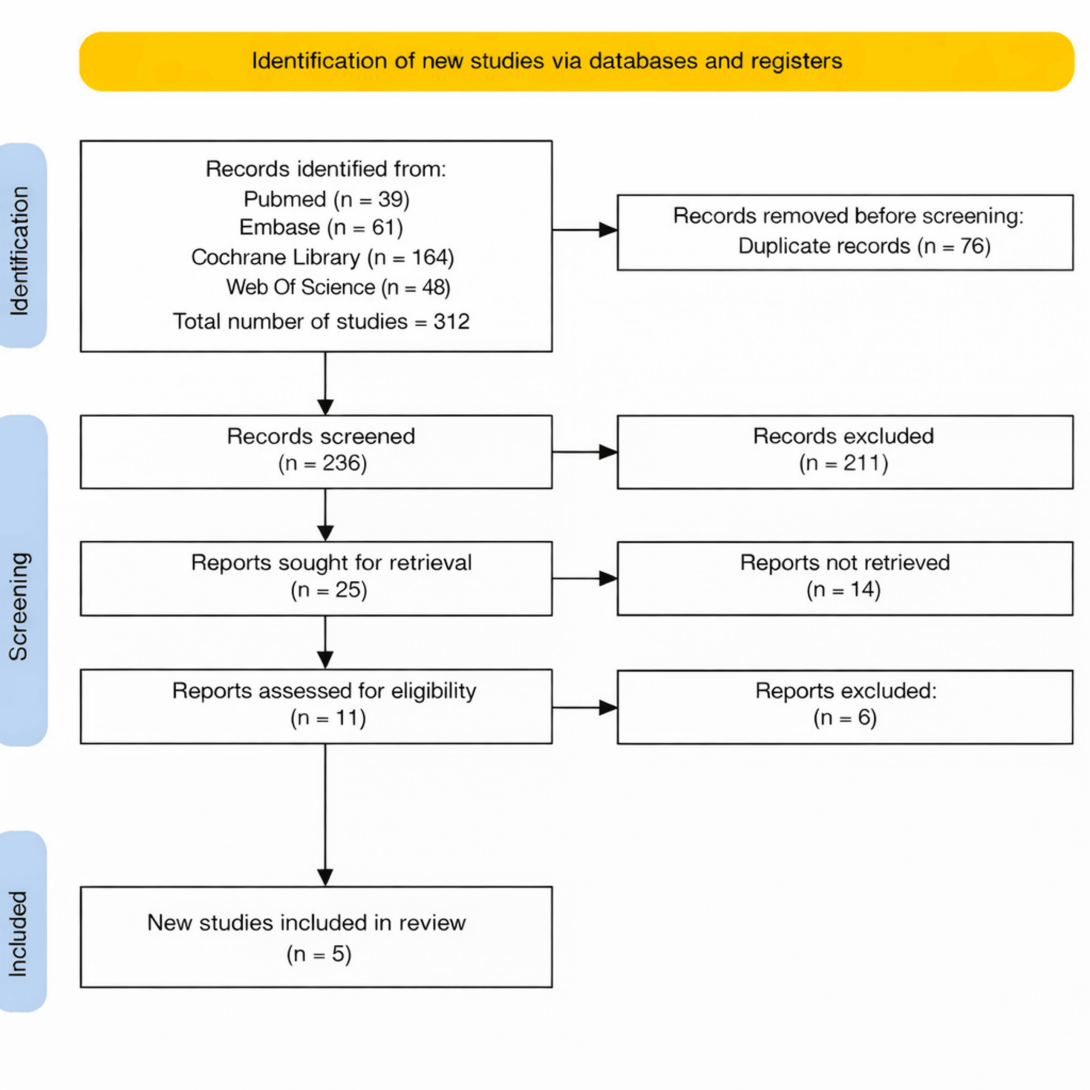
PRISMA flow diagram for the study selection process. PRISMA, Preferred Reporting Items for Systematic Reviews and Meta-Analyses

In total, five pivotal RCTs met the predefined inclusion criteria and were included [12-15,19]. The screening and inclusion steps are summarized in the PRISMA flow diagram (Figure 1).

These five trials included a total of 12,877 patients randomized to vericiguat or placebo. The key characteristics of the included trials are presented in Table 2.

**Table 2:** Characteristics of included randomized controlled trials. KCCQ, Kansas City Cardiomyopathy Questionnaire; CV, cardiovascular; HFH, heart failure hospitalization; LAV, left atrial volume; proBNP, pro-B-type natriuretic peptide

Study (author, year)	Trial name	HF phenotype	Sample size (n)	Follow-up	Intervention	Comparator	Primary outcome
Armstrong et al., 2020 [12]	VICTORIA	HFrEF (worsening)	5,050	10.8 months	Vericiguat (10 mg daily)	Placebo	Cardiovascular death or first HFH
Butler et al., 2025 [13]	VICTOR	HFrEF (stable)	~6,105	18.5 months	Vericiguat (up to 10 mg)	Placebo	Cardiovascular death or HFH
Gheorghiade et al., 2015 [19]	SOCRATES-REDUCED	HFrEF	456	12 weeks	Vericiguat (various doses)	Placebo	ProBNP change
Pieske et al., 2017 [14]	SOCRATES-PRESERVED	HFpEF	477	12 weeks	Vericiguat (various doses)	Placebo	ProBNP and LA volume
Armstrong et al., 2020 [15]	VITALITY-HFpEF	HFpEF	789	24 weeks	Vericiguat (10/15 mg)	Placebo	KCCQ physical limitation score

Three trials focused exclusively on patients with HFrEF: the trials by Armstrong et al. [12], Butler et al. [13], and Gheorghiade et al. [19], while two trials enrolled patients with HFpEF: Pieske et al. [14] and Armstrong et al. [15]. A fundamental difference among the HFrEF trials was the clinical stability of the enrolled populations. The trials by Armstrong et al. [12] and Gheorghiade et al. [19] enrolled high-risk patients with a recent worsening HF event (e.g., recent HF hospitalization or the requirement for intravenous diuretics). In contrast, the trial by Butler et al. [13] included patients with stable, chronic ambulatory HFrEF who did not have recent worsening events. This cohort was managed with highly optimized, guideline-directed medical therapy, including a high background use of ARNIs and SGLT2 inhibitors. This difference in patient risk profiles is important for interpreting the trial results.

Bias Assessment

**Figure 2:**
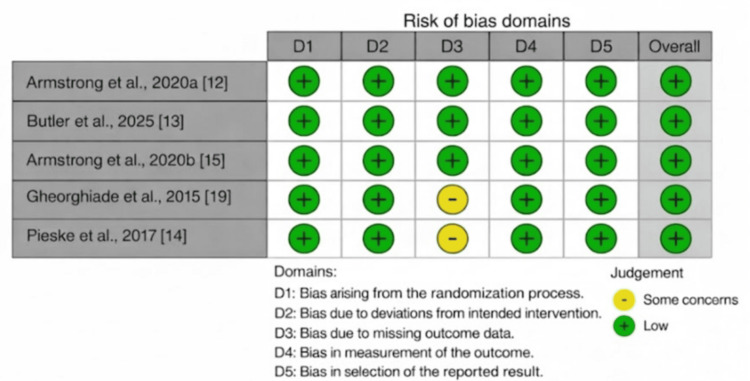
Bias assessment. Risk of bias across the five included randomized controlled trials, evaluated using the Cochrane Risk of Bias 2 (RoB 2) tool (Cochrane Collaboration, London, United Kingdom). Data derived from Armstrong et al. [12], Butler et al. [13], Gheorghiade et al. [19], Pieske et al. [14], and Armstrong et al. [15].

All included trials were multicenter, randomized, double-blind, placebo-controlled studies. The randomization processes, allocation concealment, and blinding of participants and personnel were robust. The two larger phase 3 trials [12,13] demonstrated a low risk of bias across all domains. The smaller, shorter-duration phase 2 trials [14,19] raised some concerns regarding missing outcome data due to higher dropout rates typical of early-phase studies; however, this was not judged to critically affect the overall validity of their findings (Figure 2).

Meta-analysis of efficacy outcomes

Cardiovascular Death or HF Hospitalization

In subgroup analyses stratified by HF phenotype, the effect of vericiguat differed substantially between patients with reduced and preserved ejection fraction. The HFrEF subgroup included 11,611 randomized patients across three trials (*n* = 5,050 [12]; *n* = ~6,105 [13]; *n* = 456 [19]), while the HFpEF subgroup included 1,266 patients (*n* = 477 [14]; *n* = 789 [15]).

**Figure 3:**
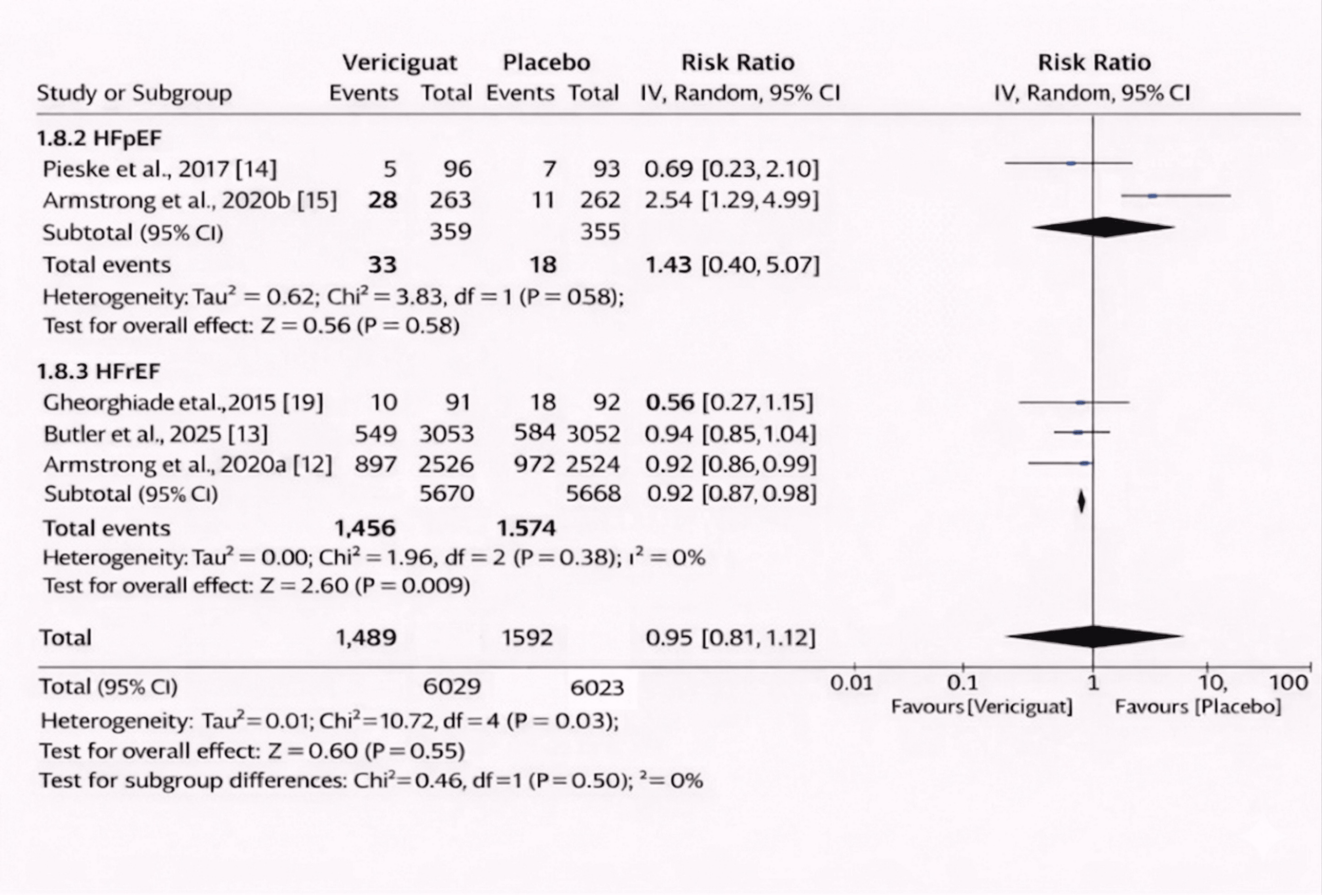
Cardiovascular death or heart failure hospitalization. Forest plot summarizing the impact of vericiguat versus placebo on the composite of CV death and HF hospitalization. Results are stratified by heart failure phenotype (HFrEF vs. HFpEF). Data are from Armstrong et al. [12], Butler et al. [13], Gheorghiade et al. [19], Pieske et al. [14], and Armstrong et al. [15]. CV, cardiovascular; HF, heart failure; HFrEF, heart failure with reduced ejection fraction; HFpEF, heart failure with preserved ejection fraction

In the HFrEF subgroup, which included 11,611 patients from the Armstrong et al. [12], Butler et al. [13], and Gheorghiade et al. [19] trials, vericiguat was associated with a significant reduction in the composite endpoint of cardiovascular death or HF hospitalization compared with placebo (RR 0.92; 95% CI, 0.87-0.98; p = 0.009), as shown in Figure 3.

In contrast, no reduction in the primary composite outcome was observed among patients with HFpEF (1,266 participants from the Pieske et al. [14] and Armstrong et al. [15] trials). The pooled estimate did not show a significant difference between vericiguat and placebo (RR 1.43; 95% CI, 0.40-5.07; *P* = 0.58), and heterogeneity was present between studies (*I*² = 74%) [14,15].

When pooling all trials regardless of ejection fraction, the overall effect of vericiguat on the primary composite outcome was not statistically significant, as shown in Figure 3.

All-Cause Mortality

**Figure 4:**
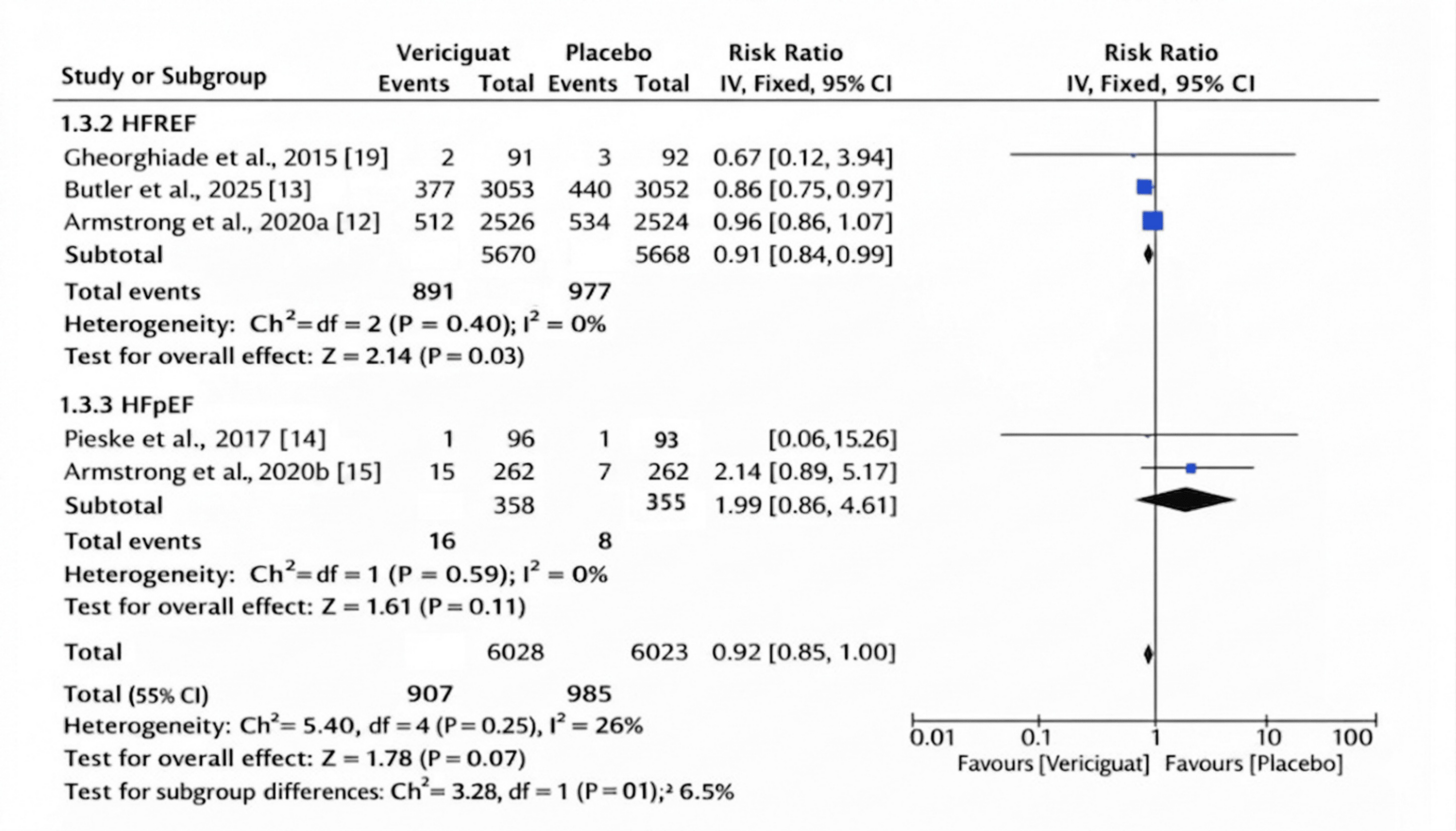
All-cause mortality. Meta-analysis of the effect of vericiguat on all-cause mortality in heart failure patients. The pooled HFrEF results are derived from Gheorghiade et al. [19], Butler et al. [13], and Armstrong et al. [12]. HFpEF data come from Pieske et al. [14] and Armstrong et al. [15]. CI, confidence interval

A similar pattern was observed for all-cause mortality. In the HFrEF subgroup, including patients from the trials by Gheorghiade et al. [19], Butler et al. [13], and Armstrong et al. [12], vericiguat was associated with a lower risk of death from any cause compared with placebo (RR 0.91; 95% CI, 0.84-0.99; *P* = 0.03), as shown in Figure 4. Results were consistent across studies, with no heterogeneity detected (I² = 0%). The trials by Butler et al. [13] and Armstrong et al. [12] accounted for most of the weight in this analysis, with Butler et al. [13] demonstrating a statistically significant mortality reduction independently.

In contrast, no significant effect on all-cause mortality was observed in the HFpEF subgroup, which included participants from the trials by Pieske et al. [14] and Armstrong et al. [15]. The pooled estimate did not demonstrate a difference between treatment groups (RR 1.99; 95% CI, 0.86-4.61; *P* = 0.11).

Because SOCRATES-REDUCED was a phase 2 dose-ranging trial not powered for clinical outcomes, a sensitivity analysis was performed restricting the primary composite outcome analysis to the two adequately powered phase 3 trials (*n* = 11,155 [12,13]). The pooled estimate was unchanged (RR 0.92; 95% CI, 0.87-0.98; *I*² = 0%), confirming that the primary efficacy conclusion is driven by the phase 3 evidence and is not artifactually influenced by inclusion of the smaller phase 2 trial.

A post hoc test for subgroup interaction between the worsening HFrEF [12] and stable HFrEF [13] populations did not reach statistical significance (Chi² = 0.46, df = 1, P = 0.50; I² = 0%). However, an interaction test based on only two trials has very limited statistical power to detect clinically meaningful effect modification, and the absence of a significant interaction should not be interpreted as evidence that the treatment effect is identical across the two populations. The clinically relevant difference between the trials, a numerically larger absolute risk reduction in the worsening HF population [12] compared with the stable ambulatory population [13], is best assessed based on the underlying trial designs, baseline event rates, and background therapy rather than on this underpowered statistical test.

When all trials were analyzed together, the overall estimate showed a borderline reduction in all-cause mortality (RR 0.92; 95% CI, 0.85-1.00; P = 0.05) [12-15,19]. The test for subgroup differences suggested a possible variation in treatment effect between the HFrEF and HFpEF populations, although this was not statistically significant

Meta-analysis of safety outcomes

Hypotension

**Figure 5:**
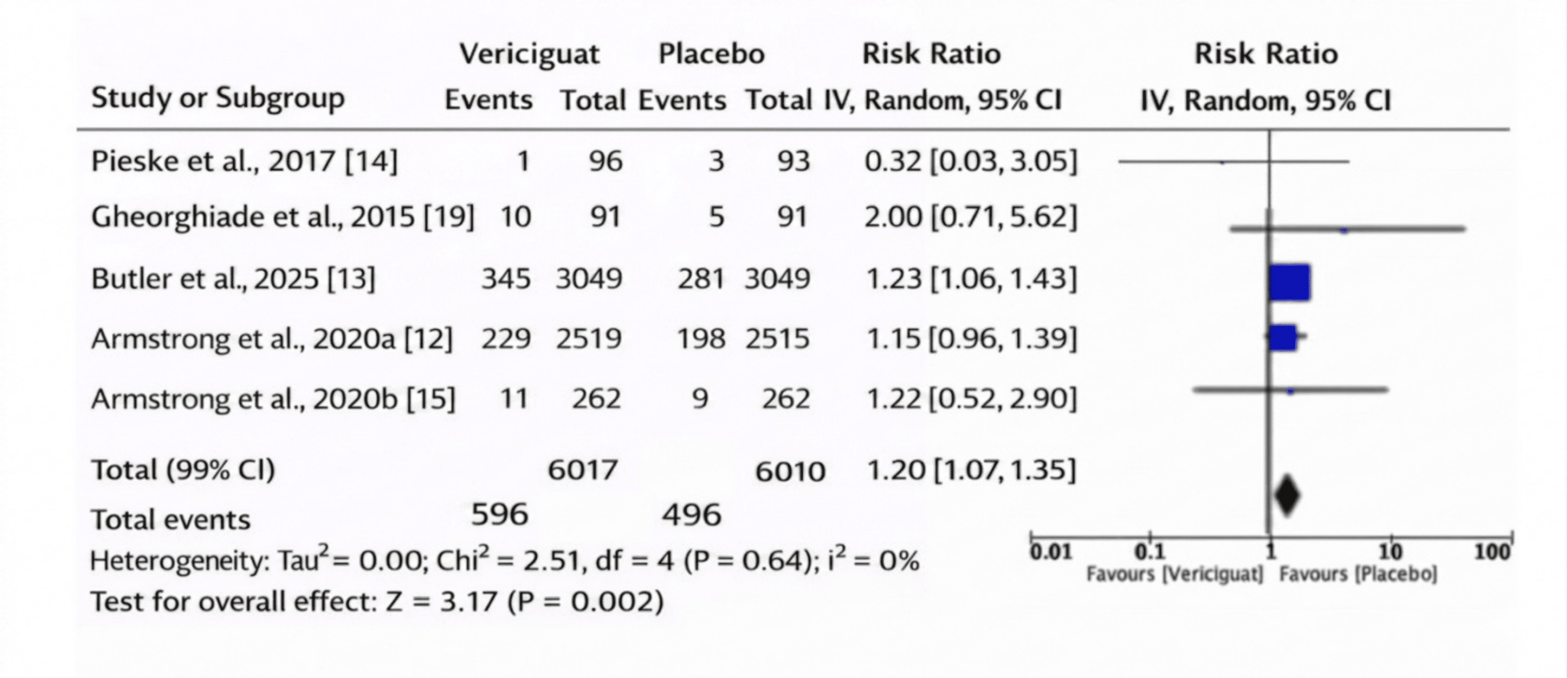
Hypotension. Forest plot summarizing the incidence of symptomatic hypotension with vericiguat compared with placebo across all five included trials [12-15,19].

Hypotension was evaluated across all five included trials [12-15,19]. The pooled analysis showed a higher risk of hypotension among patients treated with vericiguat compared with placebo (RR 1.20; 95% CI, 1.07-1.35; *P* = 0.002), as illustrated in Figure 5. The trials by Butler et al. [13] and Armstrong et al. [12] contributed the majority of the weight to this analysis.

The increased risk of hypotension was consistent across studies, with no evidence of heterogeneity (*I*² = 0%; *P* = 0.64).

Serious Adverse Events

**Figure 6:**
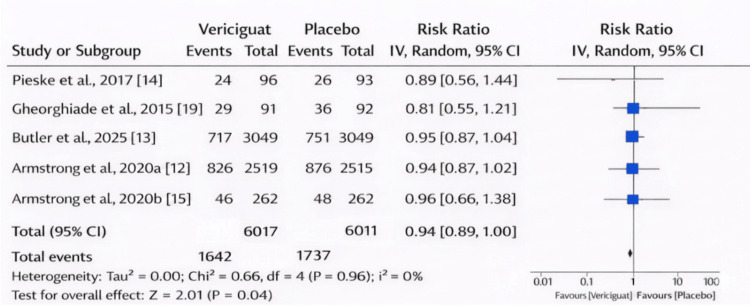
Serious adverse events. Forest plot comparing serious adverse events in patients receiving vericiguat versus placebo. Pooled data were obtained from [12-15,19].

Serious adverse events were analyzed using pooled data from all five included trials [12-15,19]. Overall, vericiguat was not associated with an increased risk of serious adverse events compared with placebo; the pooled estimate was borderline lower (RR 0.94; 95% CI, 0.89-1.00; *P* = 0.04), as shown in Figure 6.

The results were consistent across studies, with no observed heterogeneity (*I*² = 0%; *P* = 0.96).

Discussion

This updated systematic review and meta-analysis combines the complete randomized evidence, including the Butler et al. [13] trial, and helps clarify the role of vericiguat in HF. The pooled data demonstrate that vericiguat’s effectiveness is not universal across HF phenotypes but is limited to a specific subgroup of patients. The primary finding is that vericiguat is effective in reducing major cardiovascular events in patients with HFrEF [12,13,19]. However, this benefit on the composite outcome of CV death or HF hospitalization is primarily attributable to its effect in the high-risk population with a recent history of worsening HF. Vericiguat did not significantly improve this primary outcome in the broader, more stable ambulatory HFrEF population and showed no clinical benefit in patients with HFpEF [14,15]. Understanding this risk-based difference is critical for determining the appropriate clinical use of vericiguat.

HFrEF: A Tale of Two Populations

The divergent primary results of Armstrong et al. [12] and Butler et al. [13] are not contradictory; rather, they are complementary and help define the therapeutic window for vericiguat. The study by Armstrong et al. [12] enrolled a high-risk population recently decompensated by hospitalization for HF or requiring intravenous diuretics. This clinical state is marked by profound impairment of the NO-sGC-cGMP pathway that vericiguat is designed to target. In this high-risk and high-event-rate context, vericiguat provided a meaningful clinical benefit by reducing the primary composite outcome. These findings were highly consistent across studies, with no observed heterogeneity (*I*² = 0%).

In contrast, the Butler et al. [13] trial included patients with stable, chronic ambulatory HFrEF without recent worsening events. This population was treated with highly optimized, guideline-directed medical therapy, including high baseline use of ARNIs and SGLT2 inhibitors. The strong effects of these foundational therapies, established by the landmark DAPA-HF [7] and EMPEROR-Reduced [21] trials, likely attenuated the baseline event rate for HF hospitalizations to a level where an incremental benefit from vericiguat could not be statistically demonstrated.

One of the most intriguing findings from this complete body of evidence is the mortality signal observed in the study by Butler et al. [13]. The borderline all-cause mortality reduction (RR 0.91; *P* = 0.03 for HFrEF) should be interpreted with caution, as the all-cause mortality analysis was not the primary prespecified outcome in either phase 3 trial, and the overall estimate across all trials narrowly crossed the significance threshold (*P* = 0.05). Despite a neutral primary composite outcome, vericiguat was associated with fewer cardiovascular deaths (9.6% vs. 11.3% with placebo) and all-cause deaths (12.3% vs. 14.4%). Nevertheless, the observed separation between hospitalization and mortality outcomes is noteworthy. It suggests that in stable, well-treated HFrEF populations, vericiguat’s mechanism may shift from preventing acute decompensations to offering a more gradual benefit on disease progression. This mortality effect may emerge over longer follow-up periods (18.5 months in the study by Butler et al. [13] vs. 10.8 months in the study by Armstrong et al. [12]), potentially due to favorable effects on cardiac structure and reduced arrhythmic death. A subsequent study reporting lower rates of sudden cardiac death in vericiguat-treated patients supports this hypothesis [22].

HFpEF: A Consistent Lack of Benefit

The combined evidence from the Armstrong et al. [15] and Pieske et al. [14] trials shows a consistent lack of benefit for vericiguat in the HFpEF population. These studies showed no improvement in patient-reported outcomes (e.g., KCCQ score), biomarkers of cardiac stress (N-terminal pro-B-type natriuretic peptide, NT-proBNP), or structural measures (e.g., left atrial volume). This lack of effect is likely attributable to the multifactorial pathophysiology of HFpEF, which involves mechanisms such as systemic inflammation, coronary microvascular dysfunction, and myocardial stiffness that are largely independent of the NO-sGC-cGMP pathway.

Although our vericiguat-only analysis did not demonstrate a statistically significant increase in mortality in HFpEF, the direction of effect and wide confidence intervals warrant caution. Based on these findings, the available evidence does not support the use of sGC stimulators, including vericiguat, in HFpEF outside of clinical trials; there is no evidence of benefit, and the direction of effect on cardiovascular mortality warrants caution.

Positioning Vericiguat Among Current Treatment Options

This meta-analysis establishes a clear, evidence-based position for vericiguat within the current treatment landscape for HFrEF. Rather than a foundational therapy, vericiguat is best positioned as an add-on therapy for selected high-risk HFrEF patients with a recent worsening event despite optimized background treatment. This use case aligns with the 2023 Focused Update of the 2021 ESC Guidelines, which recommend vericiguat for patients following an episode of worsening HF [23].

Taken together, Armstrong et al. [12] and Butler et al. [13] support a risk-based treatment approach, with the clearest clinical benefit observed in high-risk HFrEF patients after recent worsening despite optimized background therapy. Current evidence does not support routine initiation of vericiguat in stable ambulatory HFrEF primarily to reduce the composite outcome of cardiovascular death or HF hospitalization.

A key strength of this study is that it is the first to synthesize the complete body of evidence from all five pivotal vericiguat RCTs, including Butler et al. [13]. This enables a definitive, risk-based interpretation that resolves uncertainty in previous literature. The analysis is based on high-quality, large-scale RCTs involving more than 12,000 patients, which enhances the reliability and generalizability of the findings [12-15,19].

However, several limitations must be acknowledged. First, this is a trial-level meta-analysis rather than an individual patient data (IPD) analysis, which limits the ability to explore treatment effect modifiers (e.g., NT-proBNP levels, renal function). Second, there was variability in background therapy across trials. For example, SGLT2 inhibitor use was minimal in Armstrong et al. [12] but was standard of care in Butler et al. [13]. While this limits direct comparability, it enhances the real-world relevance of the Butler et al. [13] findings. Third, as noted, the mortality findings in Butler et al. [13] were exploratory and not prespecified. Fourth, three of the five included trials were phase 2 studies not powered for hard clinical endpoints; their inclusion in pooled safety analyses and subgroup sensitivity analyses is exploratory and should be interpreted accordingly. Fifth, risk ratios (RRs) rather than hazard ratios were used for pooling. Although HR-based meta-analysis is methodologically preferred for time-to-event endpoints, binary RR calculation was used here given the availability of event count data across all trials and the practical similarity between RR and HR estimates at the observed event rates and follow-up durations. Sixth, the test for interaction between the worsening and stable HFrEF subgroups was conducted post hoc and was not prespecified in the original protocol; accordingly, this analysis should be interpreted as exploratory.

Although the overall risk of bias was low, two smaller phase 2 trials were rated as having *some concerns*, primarily due to missing outcome data [14,15,19,20]. However, sensitivity analyses excluding these studies did not change the primary conclusions, which were driven by the large, high-quality phase 3 trials.

## Conclusions

This updated systematic review and meta-analysis suggests that vericiguat provides its clearest benefit in HFrEF patients with recent worsening events. The evidence does not support routine use for the primary composite outcome in stable ambulatory HFrEF, and no clear clinical benefit was observed in HFpEF, where uncertainty remains substantial. Symptomatic hypotension was increased, while serious adverse events were not increased. These findings support a risk-stratified role for vericiguat as add-on therapy in selected high-risk HFrEF patients receiving guideline-directed medical therapy.

## Appendices

Appendix A

**Table 3:** Complete search strategies.

PubMed (searched September 10, 2025):
("heart failure"[MeSH Terms] OR "heart failure"[Title/Abstract] OR "cardiac failure"[Title/Abstract] OR "ventricular dysfunction"[Title/Abstract]) AND (vericiguat[Title/Abstract] OR "MK-1242"[Title/Abstract] OR "Verquvo"[Title/Abstract] OR "soluble guanylate cyclase stimulator"[Title/Abstract]) AND ("randomized controlled trial"[pt] OR "clinical trial"[pt])
Embase (searched September 10, 2025):
('heart failure'/exp OR 'heart failure' OR 'cardiac failure' OR 'ventricular dysfunction') AND (vericiguat OR 'MK-1242' OR 'Verquvo') AND [randomized controlled trial]/lim Cochrane Library - CENTRAL (searched September 10, 2025): ("heart failure" OR "cardiac failure") AND (vericiguat OR "soluble guanylate cyclase stimulator") - filtered to randomized controlled trials via the Cochrane RCT filter
Web of Science (searched September 10, 2025):
TS=("heart failure" OR "cardiac failure") AND TS=(vericiguat OR "soluble guanylate cyclase stimulator") - filtered to Article type; clinical trial

Appendix B

**Table 4:** GRADE summary of findings. Certainty of evidence assessed using the Grading of Recommendations Assessment, Development and Evaluation (GRADE) framework. Domains: Risk of bias assessed using Cochrane RoB 2 tool; Inconsistency assessed by I² statistic; Indirectness assessed by population, intervention, comparator, and outcome alignment; Imprecision assessed by confidence interval width and whether the CI crosses the null; Publication bias assessed qualitatively given fewer than 10 trials precluded formal funnel plot analysis

Outcome	Studies (n patients)	Risk of Bias	Inconsistency	Indirectness	Imprecision	Publication Bias	Certainty
CV Death or HF Hospitalization (HFrEF)	3 trials (n = 11,611)	Not serious (Phase 3 trials low RoB 2; Phase 2 sensitivity does not change estimate)	Not serious (I² = 0%)	Not serious (population, intervention, and outcome directly match clinical question)	Not serious (CI 0.87–0.98, does not cross 1.0)	Undetected	MODERATE — downgraded one level because the estimate pools clinically heterogeneous HFrEF populations and includes one underpowered Phase 2 trial, although Phase 3 sensitivity analysis was consistent.
All-Cause Mortality (HFrEF)	3 trials (n = 11,611)	Not serious	Not serious (I² = 0%)	Not serious	Serious (CI 0.84–0.99 is narrow but analysis was not prespecified primary outcome in either Phase 3 trial)	Undetected	MODERATE (downgraded one level for imprecision — exploratory, non-prespecified analysis)
CV Death or HF Hospitalization (HFpEF)	2 trials (n = 1,266)	Not serious	Serious (I² = 74%)	Not serious	Very serious (CI 0.40–5.07, crosses 1.0 widely; small sample)	Undetected	VERY LOW (downgraded two levels: inconsistency + imprecision)
Symptomatic Hypotension (All trials)	5 trials (n = 12,027)	Not serious	Not serious (I² = 0%)	Not serious	Not serious (CI 1.07–1.35, does not cross 1.0)	Undetected	MODERATE (downgraded one level — Phase 2 trials included, which were not powered for safety endpoints as primary outcomes)
Serious Adverse Events (All trials)	5 trials (n = 12,028)	Not serious	Not serious (I² = 0%)	Not serious	Serious (CI 0.89–1.00, upper bound touches null; p = 0.04 borderline)	Undetected	MODERATE (downgraded one level for borderline imprecision)
